# Efficacy and adverse reaction to different doses of atorvastatin in the treatment of type II diabetes mellitus

**DOI:** 10.1042/BSR20182371

**Published:** 2019-07-05

**Authors:** Hua Jiang, Hong Zheng

**Affiliations:** 1 Department of Internal Cardiovascular, The Second Hospital of Dalian Medical University, Dalian 116027, P.R. China; 2 Department of Endocrine, The Second Hospital of Dalian Medical University, Dalian 116027, P.R. China

**Keywords:** Atorvastatin, Different dose, Lipid metabolism, Type II diabetes mellitus

## Abstract

**Background:** Type II diabetes mellitus (T2DM), a persistent metabolic disorder, is primarily characterized by insulin resistance, relative insulin deficiency and dyslipidemia. Here, we aimed to investigate whether different doses of atorvastatin (ATV) affect rats with T2DM. A total of 110 Sprague–Dawley rats were successfully established as T2DM models. **Methods:** First, the total cholesterol, triglyceride (TG), high-/low-/very-low-density lipoprotein cholesterol (HDL-c/LDL-c/VLDL-c), alanine transaminase (ALT), aspartate aminotransferase (AST), blood urea nitrogen (BUN), creatinine (Cr), apolipoprotein Al (ApoA1) and apolipoprotein B (ApoB) levels in rat serum were analyzed. In addition, cholesteryl ester transfer protein (CETP) and retinol-binding protein 4 (RBP4) were also measured. Then, the incidence of adverse reactions was noted. Finally, the pathological study of liver and pancreatic tissues was performed. **Results:** Rats administered ATV at the doses of 40 and 80 mg/(kg·day) showed down-regulated TG, LDL-c, ApoB, CETP and RBP4 levels yet up-regulated HDL-c and ApoAl levels. Rats administered ATV at a dose of 80 mg/(kg·day) exhibited a higher incidence of adverse reactions and higher ALT and AST levels but lower BUN and Cr levels, which might affect liver and kidney function. Rats administered ATV at the doses of 40 and 80 mg/(kg·day) demonstrated significantly improved liver injury and pancreatic injury induced by T2DM. **Conclusion:** These data revealed that ATV could improve the lipid metabolism in T2DM rats and 40 mg/(kg·day) may serve as the optimal dose for the reduction of lipid levels and the incidence of adverse effects.

## Background

Type II diabetes mellitus (T2DM), a type of metabolic disorder, is characterized by dyslipidemia in respect to insulin resistance and relative lack of insulin. The prevalence of T2DM was estimated at 4.0% prevalence among adults in 1995; however, current statistics are estimating growth up to 7.7% by 2030 [1]. With higher prevalence of T2DM in developing countries than in developed countries, India and China account for 80% of all T2DM patients [2]. An existing report highlighted the relation of increasing T2DM prevalence to dyslipidemia [3]. As a metabolic disease, dyslipidemia is reported by abnormal levels of total cholesterol (TC) and triglycerides (TG) along with altered levels of high-density lipoprotein cholesterol (HDL-c), low-density lipoprotein cholesterol (LDL-c) and very-low-density lipoprotein cholesterol (VLDL-c). According to an epidemiological report, the current dyslipidemia sufferance percentage is 18.6% (approx. 160 million) adults, and this number has been predicted to grow [4]. The correlation between T2DM patients with a higher risk of cardiovascular diseases is prevalent. Thus, in addition to lowering their blood glucose level, controlling the blood lipid level of diabetics is also a critical step for the prevention of chronic complications.

Statins, or HMG-CoA-reductase inhibitors, are a class of cholesterol lowering drugs that function primarily as pivotal regulators in the production of cholesterol [5,6]. As effective treatment against dyslipidemia, statins (atorvastatin (ATV), rosuvastatin, simvastatin and pravastatin) have been extensively used to reduce the risk of stroke and myocardial infarction [7,8]. Additionally, the usage of statins has been well reported to attenuate the production of pro-inflammatory mediators, protect cells from oxidative damage during vascular injury, reduce platelet adhesion, activation, and thrombin generation, and ameliorate blood flow [9–11]. ATV, which functions as the most effective statin for lowering blood LDL-c, has been proven as a reducing agent for the risk of cardiovascular disease [12]. ATV can help decrease serum cholesterol levels, lower blood lipid levels and also extensively function in anti-atherogenic, anti-inflammatory and lipid metabolism in patients with T2DM [13,14]. A former study supports the effect of high-dose ATV to impair glucose tolerance due to reduced insulin sensitivity [15]. However, few studies have revealed the optimal dose of ATV in treatment of T2DM as well as the underlying mechanism by which different doses of ATV would influence T2DM treatment. Therefore, this raises concern for investigation of the effects of different ATV doses on lipid metabolism in a rat model of T2DM, thus providing useful insights for T2DM treatment.

## Materials and methods

### Model establishment

A total of 120 clean male Sprague–Dawley rats aged 4 weeks and weighing 180–220 g were acquired from the Dalian Medical University Experimental Animal Center (Dalian, Liaoning, China; Ethical number: 2018-06012). After a week of adaptive feeding with standard rat feed and deionized water, all rats were fed on a high fat and sugar diet, containing 10.0% lard, 20% sucrose, 2.5% cholesterol, 1.0% bile acid salt and 66.5% conventional feed. After 5 weeks, Streptozotocin (40 mg/kg, STZ), dissolved in a 0.1 mol/l citrate buffer (Sigma–Aldrich Chemical Company, St Louis, MO, U.S.A.), was injected intraperitoneally into the rats. Three days later, blood samples were taken from the caudal vein of the rats to measure the content of fasting plasma glucose (FPG). Successful establishment of T2DM models was illustrated by FPG ≥ 16.7 mmol/l and symptoms of polydipsia and polyuria. Apart from ten unsuccessfully established rats, the remaining 110 rats with T2DM were all participants in this experiment and continuously fed on a high sugar and fat diet.

### Animal grouping

A total of 110 rats were randomly allocated into five groups, including the diabetes group (*n*=30) and four ATV groups (10, 20, 40 and 80 mg/(kg·day), respectively) (*n*=20 for each). On the third day after T2DM model establishment, rats in the four ATV groups were given their corresponding doses of ATV by intragastric administration, while the rats in the diabetes group were given an equivalent amount of normal saline by intragastric administration, once a day for 4 weeks, with free access to feed and drink. A weekly body weight, food and water intake and excretion evaluation was conducted for each rat at the same time point. The rats were weighed after 12-h fasting over 4 weeks. Under no anesthetic conditions, the rats were fixed in a fixator to prevent them from turning over. The tail of the rat was comprehended ∼2 cm away from the needle position. The vein was pressed by thumb in order to fill the blood vessels. The tail vein was punctured using a syringe needle, from which a drop of blood was applied on the blood sugar test paper for blood glucose detection. FBG was measured using a rapid blood glucose tester (Roche Diagnostics, Mannheim, Germany).

### Biochemical analysis and enzyme-linked immunosorbent assay

After the absolute intragastric administration, the rats were in a fasting state for 12 h overnight. Next, blood (0.5 mL) sample was taken from the retro-orbital sinus of rat’s eyes after euthanasia using 2% sodium pentobarbital (30 mg/kg) via an intraperitoneal injection. The blood was centrifuged at 1610 × ***g*** at 4°C for 10 min to prepare the serum, which was then preserved at -20°C. A fully automatic biochemical analyzer was utilized to measure the levels of TC, TG, HDL-c/LDL-c/VLDL-c, alanine transaminase (ALT), aspartate aminotransferase (AST), blood urea nitrogen (BUN), creatinine (Cr), apolipoprotein Al (ApoA1) and apolipoprotein B (ApoB). Enzyme-linked immunosorbent assay (ELISA) was conducted to determine the levels of cholesteryl ester transfer protein (CETP) and retinol-binding protein 4 (RBP4). The kit was provided by Wuhan HuaMei Biotechnology Co., Ltd. (Wuhan, Hubei, China), and the microplate reader was supplied by Beijing Comsys Technology Co., Ltd. (Beijing, China).

### Adverse reaction observation

Any adverse reaction in the rats was observed, including mental disorders, vomiting, convulsions and sudden death, from which the total incidence was calculated.

### Histopathological examination

After blood collection from the eyeball, the rats were subjected to a large amount of CO_2_ for euthanasia. The abdominal cavity of rats was briefly dissected to obtain the liver, pancreas and perirenal adipose tissues, respectively. Majority of the liver tissues (5 mm from the edge of the largest lobe) and pancreatic tissues (the tail of pancreas) were fixed using 10% neutral buffered formalin, and some were stored at− 80°C for further experimentation. After fixing using formaldehyde, the liver and pancreatic tissues were then embedded, sliced, baked and stained using hematoxylin-eosin (HE), followed by observation under an optical microscope.

The obtained pancreatic tissues were fixed overnight in 4% polyformaldehyde solution (pH = 7.2) at room temperature, embedded using paraffin and prepared into 5-μm serial tissue sections. Then, the sections were positioned on a slide for staining with HE. To analyze the morphological indicators of islets, one section was selected randomly from every 6–7 sections to ensure the selection of different sections. The observations were photographed using the Olympus BX51 photograph system (Tokyo, Japan) and the area, perimeter and maximum diameter were measured and analyzed using the Image-Pro Plus 6.0, an imaging analysis software. Islet counting was conducted using an ocular micrometer and the area of each section was determined with a Vernier caliper. The area and the number of islets (per cm^2^) were calculated based on the aforementioned parameters. Shape factor (SF) was calculated as perimeter/area of the islet.

### Statistical analysis

All experimental data were processed using the SPSS 21.0 statistical software (IBM Corp. Armonk, NY, U.S.A.). Measurement data were presented as the mean ± standard deviation and were tested for normal distribution and homogeneity of variance. Comparison between multiple groups was analyzed by means of a one-way analysis of variance (ANOVA). The data with homogeneity of variance were further detected by Student-Newman-Keuls-q or Least-Significant-Difference-*t* test methods and the data with inhomogeneity of variance were analyzed by conducting a nonparametric analysis of the multiple-independent samples. Comparison of indicators at 0 and 4 w within one group was analyzed by means of a paired *t* test. Comparison of indicators at different time points was analyzed by repeated measures ANOVA, with a Tukey’s post hoc test. Enumeration data were expressed as the number of cases and comparisons among multiple groups were performed by means of the χ^2^ test. A value of *P*<0.05 was considered to be significantly different.

## Results

### ATV exerts improving effects on T2DM rats from the 3rd week

**Figure 1:**
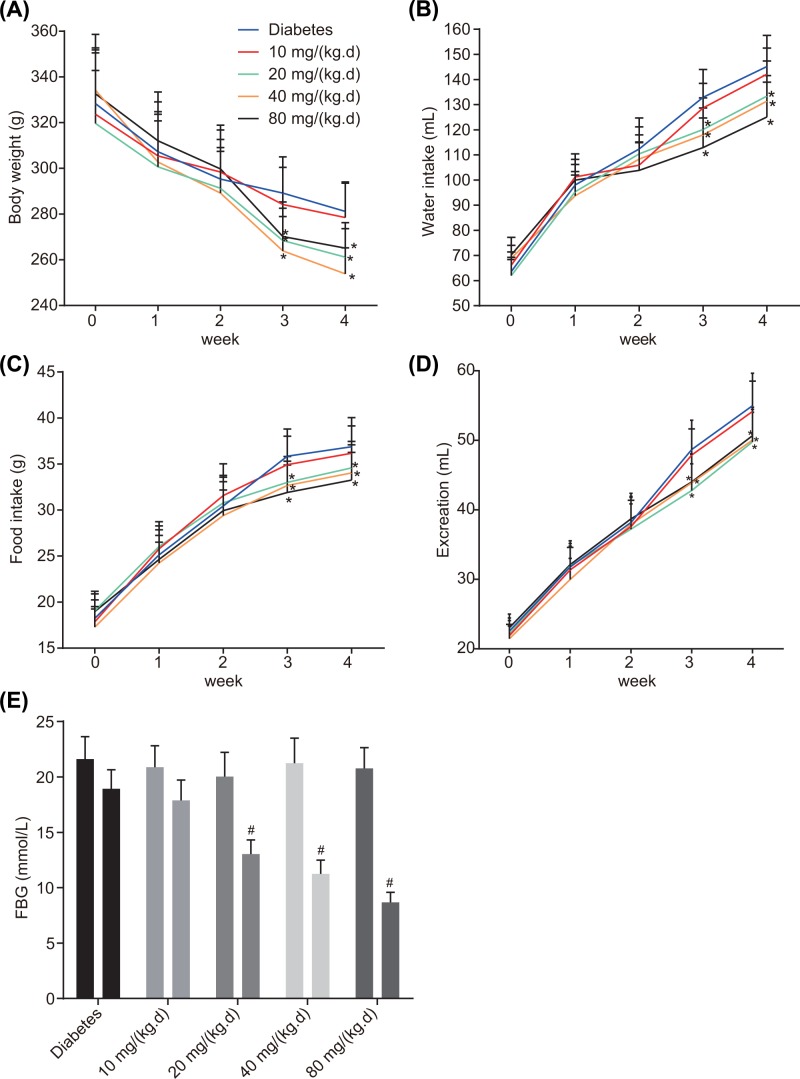
The therapeutic effects of ATV at the doses of 20, 40 and 80 mg/(kg·day) become effective from the 3rd week (**A**) Body weight of the rats in each group in 0–4 weeks of treatment; (**B**) water intake of the rats in each group in 0–4 weeks of treatment; (**C**) food intake of the rats in each group in 0–4 weeks of treatment; (**D**) excretion of the rats in each group in 0–4 weeks of treatment; (**E**) FBG in week 0 and the week 4 of treatment; diabetes group (*n*=30), ATV groups (*n*=20). Comparison of indicators at different time points was analyzed by repeated measures ANOVA, followed by Tukey’s post hoc test. *P<0.05 versus the diabetes group; #*P*<0.05 versus week 0 of treatment.

After modeling, indicators of physiological parameters (body weight, water intake, food intake, excretion and FBG) among the rats of each group during 4 weeks of treatment were measured and analyzed (Figure 1). The data showed that in the period between 0–4th week, ATV group at a dose of 10 mg/(kg.d) exhibited no significant differences compared with the diabetes group. As for ATV groups at variable doses (20, 40 and 80 mg/(kg.d)), indicators did not differ significantly during the 0, 1st and 2nd week while during the 3rd and 4th weeks of treatment they exhibited significantly decreased indicators (all *P*<0.05). In comparison with the dose of 20 mg/(kg.d), no significant difference was detected among the rats in ATV groups (40 and 80 mg/(kg.d)) during the 4 weeks of treatment. These results are depictive of the beneficial effects exerted by ATV on T2DM rats from the 3rd week at different doses (20, 40 and 80 mg/(kg.d)) with no significant difference in the effects due to different doses.

### ATV administration leads to a lower blood lipid level in a dose-dependent manner

The blood lipids of the T2DM rats in all five groups were determined using a fully automatic biochemical analyzer after 4 weeks of treatment and the results are as follows (Table 1). No significant differences were observed in the levels of TC, TG, LDL-c, VLDL-c and HDL-c between the 0 week and the 4th week in the T2DM group. In the ATV groups, after 4 weeks of treatment, TC, TG, LDL-c and VLDL-c levels decreased, while the HDL-c levels increased compared with the 0 week (*P*<0.05). After 4 weeks of treatment, compared with the diabetes group, the ATV groups at different doses of 10, 20, 40 and 80 mg/(kg·day) exhibited progressively decreased levels of TC, TG, LDL-c and VLDL-c. Further, the ATV groups at different doses of 20, 40 and 80 mg/(kg·day) demonstrated significantly reduced levels of TC and VLDL-c (*P*<0.05) while the ATV groups at the doses of 40 and 80 mg/(kg·day) showed marked decreases in levels of TG and LDL-c (*P*<0.05). In addition, all ATV groups showed progressively elevated levels of HDL-c, whereas the ATV groups at the doses of 40 and 80 mg/(kg·day) showed significantly increased HDL-c levels (*P*<0.05). These findings are illustrative of the decreased blood lipid level exhibited by the T2DM rats after treatment with ATV in a dose-dependent manner.

**Table 1:** Blood lipid indicators after 4 weeks of treatment in each group

Groups	*n*	Time points	TG (mmol/l)	TC (mmol/l)	HDL-C (mmol/l)	LDL-C (mmol/l)	VLDL-C (mmol/l)
Diabetes	30	0 W	2.15 ± 0.18	1.49 ± 0.15	1.03 ± 0.10	0. 47 ± 0.04	0.87 ± 0.09
		4 W	2.23 ± 0.18	1.45 ± 0.14	1.01 ± 0.09	0. 51 ± 0.03	0.91 ± 0.08
10 mg/(kg·day)	20	0 W	2.11 ± 0.19	1.47 ± 0.16	1.07 ± 0.09	0.45 ± 0.04	0.91 ± 0.09
		4 W	1.95 ± 0.18†	1.34 ± 0.15†	1.20 ± 0.12†	0. 39 ± 0.04†	0.76 ± 0.07†
20 mg/(kg·day)	20	0 W	2.09 ± 0.20	1.46 ± 0.15	1.05 ± 0.10	0. 46 ± 0.04	0.85 ± 0.08
		4 W	1.82 ± 0.16*†	1.31 ± 0.13†	1.24 ± 0.12†	0. 38 ± 0.04†	0.59± 0.07*†
40 mg/(kg·day)	20	0 W	2.06 ± 0.17	1.42 ± 0.13	1.11 ± 0.12	0. 48 ± 0.05	0.89 ± 0.09
		4 W	1.65 ± 0.16*†	1.15 ± 0.12*†	1.60 ± 0.16*†	0. 28 ± 0.03*†	0.50 ± 0.05*†
80 mg/(kg·day)	20	0 W	2.04 ± 0.21	1.45 ± 0.13	1.02 ± 0.11	0. 45 ± 0.04	0.86 ± 0.08
		4 W	1.47 ± 0.14*†	1.05 ± 0.15*†	1.68 ± 0.17*†	0. 25 ± 0.03*†	0.45 ± 0.04*†

Comparison at 0 and 4 W within one group was analyzed by paired *t* test. Comparison of indicators at different time points was analyzed by repeated measures analysis of variance, followed by Tukey’s post hoc test. Abbreviations: N, number; W, week. *P<0.05 versus the diabetes group. †*P*<0.05 versus the 0 W.

### ATV administration reduces lipid metabolism in a dose-dependent manner

Next, the lipid metabolism-related proteins of rats with T2DM in five groups after 4 weeks of treatment were determined, and the results are as follows (Table 2). At the 0 week, no difference was observed in the levels of lipid metabolism-related proteins in the diabetes groups compared with the 4th week. In comparison with week 0 of treatment, after 4 weeks of treatment, ApoAl levels increased, while ApoB, CETP and RBP4 levels decreased in the ATV groups at different doses of 10, 20, 40 and 80 mg/(kg·day) (*P*<0.05). All four ATV groups showed progressively elevated levels of ApoAl with the ATV groups at doses of 40 and 80 mg/(kg·day) specifically demonstrating significantly increased ApoAl levels (*P*<0.05) compared with the diabetes group. However, all ATV groups showed progressively reduced levels of ApoB, CETP and RBP4, while the ATV groups at the doses of 40 and 80 mg/(kg·day) showed prominently decreased ApoB, CETP and RBP4 levels (*P*<0.05). The results demonstrate that ATV at different doses of (10, 20, 40 and 80 mg/(kg·day)) effectively inhibits lipid metabolism with enhancement of effects in a dose-dependent manner.

**Table 2:** Lipid metabolism-related proteins after 4 weeks of treatment in each group

Group	*n*	Time points	ApoA1 (g/l)	ApoB (g/l)	CETP (µg/l)	RBP4 (µg/l)
Diabetes	30	0 W	0.75 ± 0.08	6.52 ± 0.58	4.13 ± 0.38	4.21 ± 0.43
		4 W	0.70 ± 0.08	6.73 ± 0.72	4.22 ± 0.38	4.31 ± 0.41
10 mg/(kg·day)	20	0 W	0.73 ± 0.11	6.50 ± 0.61	4.18 ± 0. 42	4.18 ± 0.42
		4 W	0.86 ± 0.10†	5.74 ± 0.53†	3.82 ± 0.39†	3.86 ± 0.34†
20 mg/(kg·day)	20	0 W	0.76 ± 0.07	6.46 ± 0.63	4.11 ± 0.39	4.15 ± 0.40
		4 W	0.89 ± 0.09†	5.58 ± 0.52†	3.74 ± 0.29†	3.75 ± 0.39†
40 mg/(kg·day)	20	0 W	0.74 ± 0.07	6.49 ± 0.59	4.15 ± 0.4	4.14 ± 0.39
		4 W	0.96 ± 0.12*	4.52 ± 0.45*	1.97 ± 0.18*	2.27 ± 0.19*
80 mg/(kg·day)	20	0 W	0.78 ± 0.08	6.43 ± 0.57	4.16 ± 0.42	4.19 ± 0.42
		4 W	1.08 ± 0.12*	3.37 ± 0.36*	1.48 ± 0.15*	1.78 ± 0.16*

Comparison at 0 and 4 W within one group was analyzed by paired *t* test. Comparison of indicators at different time points was analyzed by repeated measures analysis of variance, followed by Tukey’s post hoc test. Abbreviations: N, number; W, week. *P<0.05 versus the diabetes group. †*P*<0.05 versus the 0 W.

### ATV administration at a dose of 80 mg/(kg·day) obviously affects liver and kidney function

The liver and kidney function of the T2DM rats in all groups was analyzed after 4 weeks of treatment and the results are as follows (Table 3). At the 0 week, no difference was observed in the indicators of liver and kidney function in each group. In comparison with treatment at the 0 week, after 4 weeks of treatment, up-regulated levels of ALT and AST were observed with down-regulated levels of Cr and BUN in the diabetes group and all four ATV groups (*P*<0.05). The ATV groups at variable doses (10, 20 and 40 mg/(kg·day)) demonstrated elevated levels of ALT and AST with reduced levels of BUN and Cr, as the differences were not significant, the effects on liver and kidney function were minor (*P*>0.05) compared with the diabetes group. However, the ATV group at a dose of 80 mg/(kg·day) showed significantly enhanced levels of ALT and AST with markedly decreased levels of BUN and Cr (all *P*<0.05), which was conclusive of noticeable effects on liver and kidney function. Therefore, the results demonstrated that ATV at a dose of 80 mg/(kg·day) exhibits significant effects on liver and kidney function.

**Table 3:** Indicators of liver and kidney function after four weeks of treatment in each group

Group	*n*	Time points	ALT (U/l)	AST (U/l)	Cr (µmol/l)	BUN (mmol/l)
Diabetes	30	0 W	29.76 ± 2.67	26.04 ± 2.45	7.96 ± 0.92	126.53 ± 11.97
		4 W	24.26 ± 4.59†	26.89 ± 3.58†	8.97 ± 0.79†	142.61 ± 12.62†
10 mg/(kg·day)	20	0 W	30.25 ± 3.04	25.21 ± 2.38	7.92 ± 0.84	128.29 ± 12.45
		4 W	44.87 ± 4.57†	37.12 ± 3.65†	6.83 ± 0.68†	99.79 ± 11.44†
20 mg/(kg·day)	20	0 W	29.02 ± 3.05	25.63 ± 2.56	7.57 ± 0.79	123.29 ± 11.58
		4 W	45.15 ± 4.52†	37.94 ± 3.83†	6.78 ± 0.73†	95.83 ± 10.56†
40 mg/(kg·day)	20	0 W	31.23 ± 3.16	27.35 ± 2.81	7.79 ± 0.87	120.98 ± 12.15
		4 W	46.08 ± 4.64†	38.26 ± 3.59†	6.63 ± 0.71†	93.52 ± 11.25†
80 mg/(kg·day)	20	0 W	31.78 ± 2.98	26.83 ± 2.69	7.98 ± 0.96	130.27 ± 12.69
		4 W	53.02 ± 5.25*†	48.24 ± 5.02*†	3.01 ± 0.36*†	63.76 ± 7.16*†

Comparison at 0 and 4 W within one group was analyzed by paired *t* test. Comparison of indicators at different time points was analyzed by repeated measures analysis of variance, followed by Tukey’s post hoc test. Abbreviations: N, number; W, week. *P<0.05 versus the diabetes group. †*P*<0.05 versus the 0 W.

### ATV administration at a dose of 80 mg/(kg·day) results in higher incidence of adverse reactions

Adverse reactions in rats with T2DM in the ATV groups at different doses were analyzed, and the results are as follows (Table 4). In comparison with the diabetes group, the total incidence rate of adverse effects in the ATV group at a dose of 10 mg/(kg·day) was similar with the total incidence rate in the ATV groups at the doses of 20 and 40 mg/(kg·day) reported to be mildly elevated (20% vs 13.3), while the ATV group at a dose of 80 mg/(kg·day) exhibited the highest total incidence rate (*P*<0.05). To conclude, gathered evidence supports the use of ATV at a relatively low dose to avoid adverse effects.

**Table 4:** Adverse reaction of rats with T2DM among different dosage of ATV groups

Groups	*n*	Mental disorder	Vomit	Hyperspasmia	Sudden death	Incidence
Diabetes	30	2	1	0	1	13.3%
10 mg/kg/day	20	2	1	0	0	15.0%
20 mg/kg/day	20	2	1	1	0	20.0%
40 mg/kg/day	20	2	1	1	0	20.0%
80 mg/kg/day	20	3	2	2	1	40.0%

Data were analyzed by χ^2^ test. Abbreviation: N, number of cases.

### ATV administration at doses of 40 and 80 mg/(kg·day) improves the T2DM-induced liver injury

**Figure 2:**
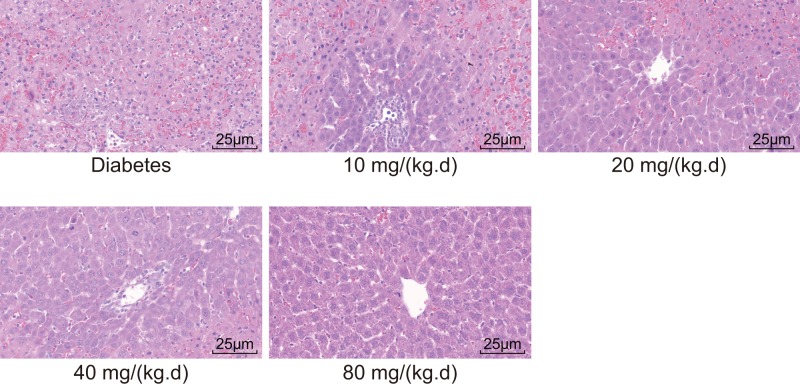
The rats administrated ATV at the doses of 40 and 80 mg/(kg·day) show improved liver injury induced by T2DM (×400)

The morphological changes in the liver tissues of T2DM rats in all five groups were measured by conducting HE staining after 4 weeks of treatment. After HE staining, the cytoplasm of liver cells in pathological tissue presented in red, while the nucleus was bluish violet (Figure 2). The diabetes group exhibited an obscure boundary of the hepatic lobule with disordered and loose hepatic cords structures. Majority of the liver cells appeared swollen, with some shrinkage and steatosis. Vacuoles of different sizes existed in the cytoplasm. In the ATV group at a dose of 10 mg/(kg·day), only a small number of cloudy and swollen liver cells were observed with few lipid droplets becoming vacuolized, which was better than the diabetes group. In the ATV group at a dose of 20 mg/(kg·day), some liver cells showed signs of cloudy swelling while only a few instances of hepatic steatosis and vacuolization were observed. Moreover, the hepatic cords in the ATV group at a dose of 20 mg/(kg·day) were more regular with a lesser obscure lobular architecture compared with the diabetes group. In the ATV groups at the doses of 40 and 80 mg/(kg·day), the lobular architecture was quite clear, the hepatic cords were orderly arranged with intact liver cell morphology. Additionally, no cloudy swelling or lipid droplets were observed. These findings signify that ATV at doses of 40 and 80 mg/(kg·day) effectively ameliorates T2DM-induced liver injury.

### ATV administration at doses of 40 and 80 mg/(kg·day) improves the T2DM-induced pancreatic injury

**Figure 3:**
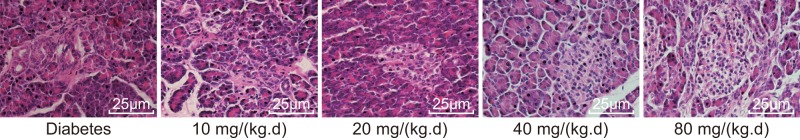
The rats administrated ATV at the doses of 40 and 80 mg/(kg·day) exhibit improved pancreatic injury induced by T2DM (×400)

Finally, the morphological changes in the pathological tissues of the T2DM rat pancreas after 4 weeks of treatment in all five groups were evaluated. As shown in Figure 3, in the diabetes group, the number and volume of pancreatic islets decreased, accompanied by the presence of several vacuoles in the islets. The boundary of the pancreatic islets was also incomprehensible and the islet cells were atrophied. However, compared with the diabetes group, in the ATV groups at different doses of 10, 20, 40 and 80 mg/(kg·day), the structure of the islet appeared intact with more numbers, and lesser pycnosis. These changes were evident in the ATV group at a dose of 40 mg/(kg·day). The morphological indicator changes of the islets are shown in Table 5, which suggested that in comparison with the diabetes group, the diameter of the islet, the area and number of the islet (per cm^2^), and the SF indicators were improved in the ATV groups with emphasis on the statistically significant differences in the ATV groups at doses of 40 and 80 mg/(kg.d). Thus, the results manifest that ATV at the doses of 40 and 80 mg/(kg·day) effectively improve pancreatic injury induced by T2DM.

**Table 5:** Morphological indicators of islet in each group

Group	*n*	Maximum diameter (μm)	Islet area/cm^2^ (μm^2^/cm^2^)	Islet number/cm^2^	Shape factor
Diabetes	30	100.75 ± 8.97	5297.13 ± 477.02	0.93 ± 0.55	0.073 ± 0.014
10 mg/(kg·day)	20	102.74 ± 9.11	5305.45 ± 473.98	1.25 ± 0.55	0.068 ± 0.011
20 mg/(kg·day)	20	107.74 ± 8.41	5324.09 ± 513.42	1.30 ± 0.57	0.066 ± 0.009
40 mg/(kg·day)	20	184.66 ± 17.61*	20536.68 ± 1913.52*	5.35 ± 0.81*	0.025 ± 0.004*
80 mg/(kg.day)	20	176.23 ± 16.26*	19817.09 ± 1853.95*	5.10 ± 0.72*	0.030 ± 0.005*

Comparison among multiple groups was analyzed by one-way analysis of variance, followed by Tukey’s post hoc test. Abbreviation: N, number. *P<0.05 versus the diabetes group

## Discussion

T2DM, the most prevalent metabolic disorder worldwide, is primarily caused due to obesity and insulin resistance, and has become an unprecedented epidemic affecting both developing and developed countries [16]. Meanwhile, abnormal lipid metabolism has been regarded to be among the most important risk factors of atherosclerosis and cardiovascular, cerebrovascular and microvascular disease [17]. Therefore, active treatments for T2DM patients to prevent cardiovascular and metabolic diseases and control blood lipid levels are of great urgency in the clinical setting as well as for researchers. Presently, ATV has been clinically approved as a cholesterol-lowering agent for T2DM patients. Research conducted to evaluate the beneficial effects of ATV flagged the superior effect of ATV on reducing TC, TG and LDL-c levels compared with other HMG-CoA-reductase inhibitors, such as rosuvastatin and simvastatin [8]. Essentially, an existing study concluded that ATV could significantly lower creatine kinase levels without affecting the liver transaminase levels [18]. In addition, ATV appears optimal in respect to adverse reactions (2–9%) and safety [19]. However, side effects due to ATV have been prevalent in patients, such as muscle pain, digestive system disorders and increased liver enzyme concentration [20]. Thus, this study was conducted to investigate the efficacy and safety of different ATV doses on lipid metabolism in a rat model with T2DM. Cooperatively, our data supports the usage of ATV at a dose of 40 mg/(kg·day) due to its optimal effect.

Initially, we found that ATV at the doses of 20, 40 and 80 mg/(kg·day) led to significantly reduced TC and VLDL-c levels, while ATV at the doses of 40 and 80 mg/(kg·day) exhibited markedly decreased TG, LDL-c, ApoB, CETP and RBP4 levels along with elevated HDL-c and ApoAl levels in T2DM rats, highlighting the significance of the capability of using high-ATV doses to ameliorate blood lipid metabolism. ATV has been demonstrated to decrease inflammation and reduce urinary albumin excretion [21]. A study focusing on the treatment of diabetic nephropathy using ATV, flagged the usage of 20 mg/(kg·day) to ameliorate lipid and glucose metabolic disorders and protect vascular function [22]. Moreover, a study illustrated the usage of ATV at a dose of 20 mg/day to contribute to post prandial insulinemia and glycemia in patients with myocardial infarction at 12 months [23].

Meanwhile, our findings demonstrated the administration of 40 mg/(kg·day) ATV to significantly reduce the risk of vascular disease in T2DM patients and reduce the TG, TC, LDL-c levels while simultaneously up-regulating HDL-c levels, thereby regulating the area of atheromatous plaque. High-dose ATV treatment would be comprehended as a contributing risk for new-onset T2DM [24]. Additionally, 80 mg/(kg·day) ATV led to a higher incidence of adverse reactions with higher ALT and AST levels, but lower BUN and Cr levels. In contrast, ATV at doses of 40 and 80 mg/(kg·day) were depictive of improved liver and pancreatic injury, thereby suggesting the use of ATV as a dosage of 40 mg/(kg·day) to have the greatest effect on lipid metabolism in a rat model of T2DM. A recent study has demonstrated that 10 mg ATV, due to its improved cardiovascular outcomes and low costs, has been ascertained as the primary preventive intervention for patients with T2DM in Belgium compared with patients who have no access to treatment [25]. Further, doubling the dose to 40 mg/(kg·day) can significantly ameliorate lipid metabolism and relax the endothelium and further reduce the area of atheromatous plaque and the thickness of endothelium, thereby effectively functioning to prevent atherosclerosis in T2DM patients [26]. Besides, the difference regarding treatment efficacy between full-dose and half-dose administration of ATV is minimal, with no severe adverse reactions from either [27].

It has been shown that after treatment with 10-mg ATV, the blood glucose level and insulin resistance in patients suffering from T2DM with dyslipidemia did not differ greatly, possibly because of the poor orientation of lipid metabolism in patients and also due to the inconsistency in the ATV dose [28]. In addition, a previous study based on the T2DM model ascertained the tendency of ATV to inhibit T2DM progression [29]. Our study was performed in consistency with the following novelties different from previous studies. Initially, we investigated the effect of ATV on lipid metabolism in T2DM. Second, this study, with different doses of ATV, has elucidated the relation between ATV dose and T2DM, which highlighted the dose-dependent effect. Third, apart from the effect of ATV on lipid metabolism, the present study also investigated its role in regard to liver and kidney function with emphasis on the safety of ATV. In addition, our researchers will make significant efforts to explore other mechanisms affecting T2DM in the future.

In summary, ATV can improve the lipid metabolism in rats with T2DM by means of regulation of the levels of oA1,1ApoB, CETP and RBP4. Additionally, the 40 mg/(kg·day) dose was concluded as the optimal dose of ATV to lower blood lipid levels and reduce the incidence of adverse reactions in rats with T2DM. The present study is expected to provide an insight as the theoretical basis for the existing relationship between ATV dose and T2DM treatment and prevention. However, the effects of loss of body weight on blood lipid levels cannot be neglected, which will be further explored by using rats of different weights. Besides, the therapeutic effects of ATV validated in our study are based on a 4-week treatment period, suggesting that usage of ATV for a short period can decrease blood glucose concentration while long-term treatment might be comprehended differently. Herein, further investigations are required to uncover the possible mechanism. In addition, the underlying mechanism for how ATV ameliorates liver cell steatosis and pancreatic damage remains elusive. Therefore, these limitations should be comprehended in future studies with a larger sample size and more advanced technologies.

## Abbreviations

ALT: alanine transaminase
ANOVA: a one-way analysis of variance
ApoB: apolipoprotein B
AST: aspartate aminotransferase
ATV: atorvastatin
BUN: blood urea nitrogen
CETP: cholesteryl ester transfer protein
Cr: creatinine
FPG: fasting plasma glucose
HDL-c: high-density lipoprotein cholesterol
HE: Hematoxylin-eosin
HMG-CoA: 3-hydroxy-3-methy glutary coenzyme A reductase
LDL-c: low-density lipoprotein cholesterol
RBP4: retinol-binding protein 4
SF: shape factor
TC: total cholesterol
TG: triglyceride
T2DM: type II diabetes mellitus
VLDL-c: very-low-density lipoprotein cholesterol
